# Relationship between semiquantitative ^18^F-fluorodeoxyglucose positron emission tomography metrics and necrosis in classical Hodgkin lymphoma

**DOI:** 10.1038/s41598-019-47453-5

**Published:** 2019-07-30

**Authors:** X. U. Kahle, F. M. Montes de Jesus, T. C. Kwee, T. van Meerten, A. Diepstra, S. Rosati, A. W. J. M. Glaudemans, W. Noordzij, W. J. Plattel, M. Nijland

**Affiliations:** 1 Department of Hematology, University of Groningen, University Medical Center Groningen, Groningen, The Netherlands; 2 Department of Nuclear Medicine and Molecular Imaging, University of Groningen, University Medical Center Groningen, Groningen, The Netherlands; 3 Department of Radiology, University of Groningen, University Medical Center Groningen, Groningen, The Netherlands; 4 Department of Pathology and Medical Biology, University of Groningen, University Medical Center Groningen, Groningen, The Netherlands

**Keywords:** Prognostic markers, Cancer imaging, Hodgkin lymphoma

## Abstract

Semiquantitative ^18^F-fluoro-2-deoxy-D-glucose positron emission tomography (^18^F-FDG PET) parameters have been proposed as prognostic markers in classical Hodgkin lymphoma (cHL). In non-Hodgkin lymphoma necrosis as assessed by ^18^F-FDG PET or computed tomography (CT) (necrosis^visual^) correlates with an adverse prognosis. We investigated whether semiquantitative ^18^F-FDG PET metrics correlate with necrosis^visual^, determined the incidence of necrosis^visual^ and explored the prognostic impact of these factors in cHL. From 87 cHL cases treated with ABVD, (escalated) BEACOPP or CHOP chemotherapy between 2010 and 2017, 71 had both a NEDPAS/EARL accredited ^18^F-FDG PET and a contrast enhanced CT scan. Semiquantitative ^18^F-FDG PET parameters were determined using Hermes Hybrid 3D software. Necrosis^visual^, defined by photopenic tumor areas on ^18^F-FDG PET and attenuation values between 10 and 30 Hounsfield units (HUs) on CT, was assessed blinded to outcome. Univariate Cox regression survival analyses of progression free survival (PFS) were performed. Necrosis^visual^ was observed in 18.3% of cHL patients. Bulky disease (tumor mass >10 cm in any direction) (*P* = 0.002) and TLG (*P* = 0.041) but no other semiquantitative parameters were significantly associated with necrosis^visual^. In exploratory univariate survival analysis for PFS the covariates IPS, bulky disease, MTV and TLG were prognostic, while necrosis^visual^ was not.

## Introduction

Classical Hodgkin lymphoma (cHL) is a B-cell neoplasm characterized by a minority of malignant Hodgkin-Reed Sternberg (HRS) cells in an inflammatory background^1^. Patients with cHL have an good prognosis with a long-term survival probability of over 90%. Nonetheless, about 10% of patients with early-stage (stage I-II) and 20–30% with advanced stage disease (III-IV) according to the Ann Arbor classification^2^, are refractory or relapse after first line therapy^3^. Therefore, it is important to improve prognostic and predictive models, in order to optimize treatment results and reduce therapy-related toxicity. Several prognostic models are used for clinical risk stratification, including the international prognostic score (IPS) for advanced stage disease, the European Organization for Research and Treatment of Cancer (EORTC) score or the German Hodgkin Study Group (GHSG) classification for early stage disease. New prognostic biomarkers for cHL include serum levels of the Thymus and Activation Regulated Chemokine (TARC)^4–8^ and tissue gene expression profiles (GEP)^9,10^. Although promising, these markers are currently not routinely used to guide treatment.

Imaging markers evaluated in cHL have focused on metabolic tumor volume (MTV) as quantified by ^18^F-fluoro-2-deoxy-D-glucose positron emission tomography (^18^F-FDG PET)^11–13^. However, results on the prognostic value of MTV in cHL are inconsistent^14–16^. Although efforts to standardize semiquantitative measurements have been made^17,18^, many study results are not comparable and so far no uniform cut-off values for the interpretation of parameters such as MTV have been established^19^. The prognostic potential of total lesion glycolysis (TLG), defined as the product of mean standard uptake value (SUV_mean_) and MTV, was recently shown in relapsed or refractory cHL^20^ and early unfavorable cHL^21^.

Tumor necrosis can be observed in the center of fast growing tumor lesions, illustrating the proposed underlying pathophysiological mechanism, in which tumors outgrow the existing nutrient and oxygen supplies^22,23^. In non-Hodgkin lymphoma (NHL), necrosis as assessed by magnetic resonance imaging (MRI) and computed tomography (CT) correlates with high tumor volume and high clinical risk scores^24^. Tumor necrosis as assessed using ^18^F-FDG PET scans and CT imaging (necrosis^visual^) has been shown to be an adverse prognostic factor in NHL^25–28^. Previously, a study of 76 cHL patients with a thoracic mass treated with MOPP-like regimens, reported necrosis as assessed with CT scans in 21% of cases, but no significant impact on length of remission or overall survival (OS)^29^. However, with the introduction of ^18^F-FDG PET into clinical routine, whole-body staging has significantly improved^30^, warranting for a new investigation into the prognostic impact of necrosis^visual^. In contrast to novel semiquantitative imaging markers, necrosis^visual^ can be easily detected using ^18^F-FDG PET or CT scans and has a dichotomous outcome.

The aims of the current study were (I) to determine the incidence of necrosis^visual^ in cHL, (II) to evaluate its correlation with semiquantitative ^18^F-FDG-PET metrics, and (III) to explore the prognostic impact of these factors with regard to outcome.

## Methods

### Study design and case selection

For this retrospective single center study a consecutive series of 87 patients with histologically confirmed cHL according to WHO classification 2008^31^ were initially identified in the electronic healthcare database of the University Medical Center Groningen (UMCG). Cases of nodular lymphocyte predominant HL, composite lymphoma and Hodgkin-like immuno-deficiency-associated lymphomas were excluded. Patients were treated between 2010 and 2017 according to ESMO guidelines^32,33^. Patient with limited stage disease (stage I-II) received doxorubicin, bleomycin, vinblastine, and dacarbazine (ABVD) with radiotherapy, whereas patients with advanced stage disease (stage III-IV) were treated with either full course ABVD or escalated bleomycin, etoposide, doxorubicin, cyclophosphamide, vincristine, procarbazine, prednisone (escBEACOPP) (Supplementary Tables 1 and 2). Four patients with severe pre-existent lung disease were treated with cyclophosphamide, doxorubicin, vincristine, prednisone (CHOP) to prevent bleomycin-mediated lung toxicity^34^.

**Figure 1:**
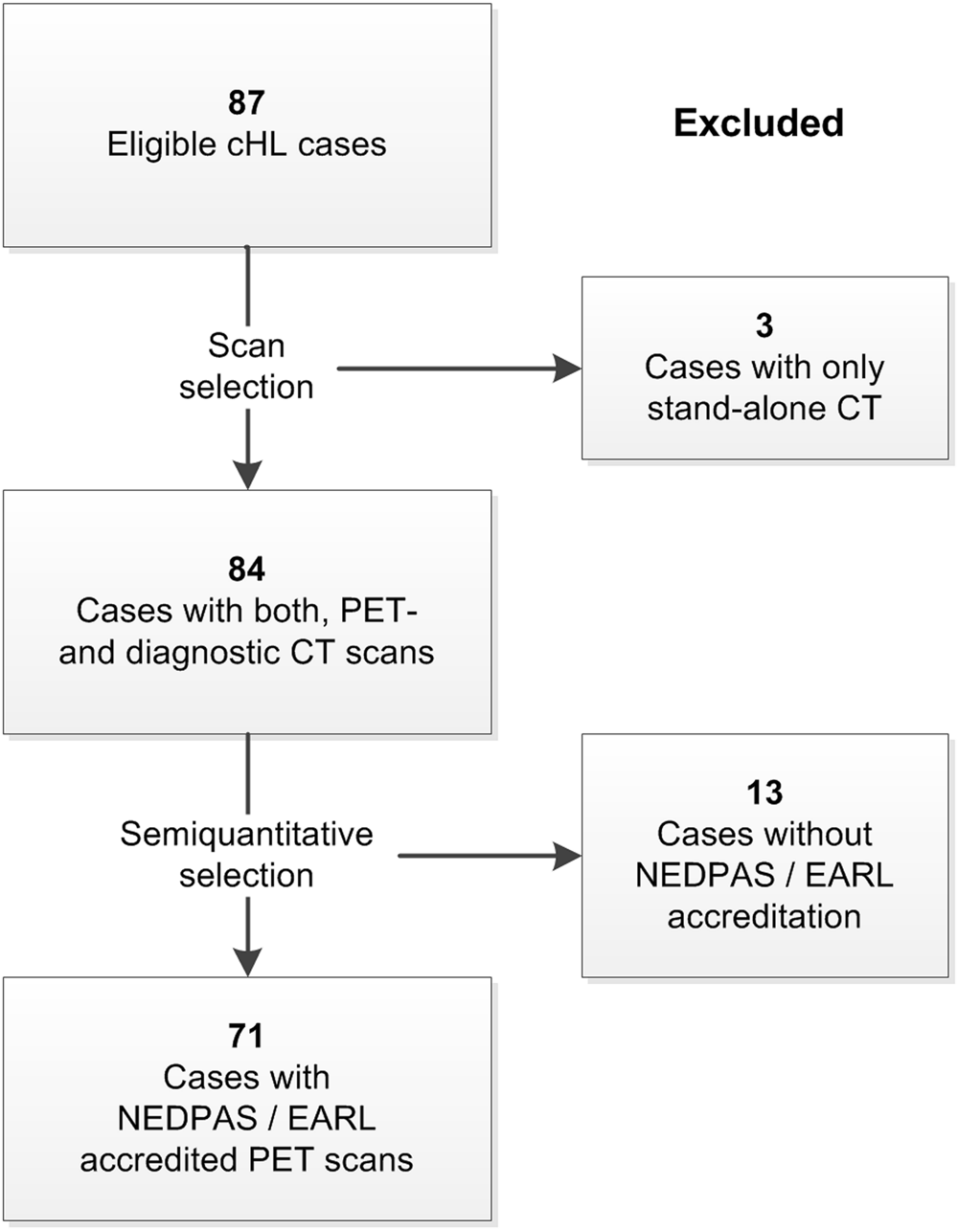
Flow chart illustrating the case selection procedure for this study; cHL = classical Hodgkin lymphoma; PET = positron emission tomography; CT = computed tomography.

Of the 87 patients 3 had only stand-alone diagnostic CT scans and were excluded from further analysis. In 13 cases acquisition methods of ^18^F-FDG PET/CT scans did not comply with guidelines specified by the “Netherlands protocol for standardization of ^18^F-FDG whole-body PET studies in multi-center trials” (NEDPAS)^35^ or “European Association of Nuclear Medicine Research Ltd.” (EARL) protocols^36^. Finally, a total of 71 cases were eligible for analysis (Fig. 1). First and second order semiquantitative metrics were analyzed. Patients were stratified according to international prognostic score (IPS)^37^. End of treatment response was assessed by ^18^F-FDG PET/CT scan. For PET scans a Deauville score of ≤3 was interpreted as complete metabolic response in the absence of new lesions. Treatment response was classified as complete remission (CR), partial response (PR), stable disease (SD), or progressive disease (PD) according to Lugano criteria^38^. All cases with relapse were histologically confirmed. Follow-up was registered until February 2018. According to Dutch regulations, no medical ethical committee approval was required for this retrospective, observational study. A waiver was obtained from the medical ethics committee of the UMCG on November 13^th^ 2018. All data were coded before analysis.

### ^18^F-FDG PET imaging

Integrated ^18^F-FDG PET/CT images were acquired on a Biograph mCT (Siemens Medical Systems, Knoxville, TN, USA) after a minimum fasting time of 6 h. Whole body images (from the base of the skull to the mid-thigh) were acquired 60 minutes after intravenous administration of 3MBq/kg ^18^F-FDG. PET image acquisition for patients between 60 and 90 kg bodyweight was performed in 7 bed positions of 2 minute emission scans. Patients with bodyweight below 60 were imaged for 1.5 minute and above 90 kg for 3 minutes per bed position. Integrated ^18^F-FDG PET/CT images were corrected for scatter and attenuation based on CT information and automatically fused through three-dimensional fusion software (Siemens). Raw data were reconstructed through ultra-high definition (Siemens).

### Semiquantitative analyses

Semiquantitative analyses were performed using the “Tumor Finder” application and the Hermes Hybrid 3D (Hermes Medical Solutions AB, Stockholm, Sweden) software, as described previously^39^. Briefly, NEDPAS/EARL accredited ^18^F-FDG PET and low-dose CT files were loaded from the UMCG electronic database. A spherical 3 cm^3^ volume of interest (VOI) over the right lobe of the liver was used as a reference according to PERCIST 1.0 criteria^40^ and the “Tumor Finder” application automatically selected all VOIs with uptake ≥ 1.5 × mean SUV + 2 standard deviations (SDs) of this reference VOI. Volumes of high physiological uptake, not suspected for lymphoma were manually removed, while volumes suspected for disease involvement, but not automatically selected by the “Tumor Finder” application were selected manually using a threshold of ≥2.5 SUV corrected for body weight (SUVbw). Semiquantitative measurements included maximum standardized uptake value (SUV_max_), SUV_peak_, SUV_mean_, MTV and TLG. SUV_max_ was defined as the SUV of the maximum intensity voxel within a region of interest (ROI), SUV_peak_ as the spherical 1 cm^3^ VOI within the borders of a lesion with the highest average SUV, SUV_mean_ as the average of SUV within a ROI, MTV as the total metabolically active volume of segmented tumor and TLG as the product of SUV_mean_ × MTV (summed over all lesions). To be representative of the entire lymphoma SUV_max/peak/mean_ are reported here as means calculated across all lesions.

### CT imaging

Full-dose, intra-venous contrast enhancement, diagnostic CT scans of neck, chest and abdomen were acquired as part of an integrated ^18^F-FDG PET/CT. CT scans were acquired using different multidetector row (≥16-slice) CT scanners (Somatom Series, Siemens Healthineers, Erlangen, Germany). Presence of bulky disease was defined with lymphomatous lesion with a diameter of more than 10 cm in any direction, as assessed by full-dose, intra-venous contrast enhancement, diagnostic CT imaging.

### Assessment of necrosis^visual^

**Figure 2:**
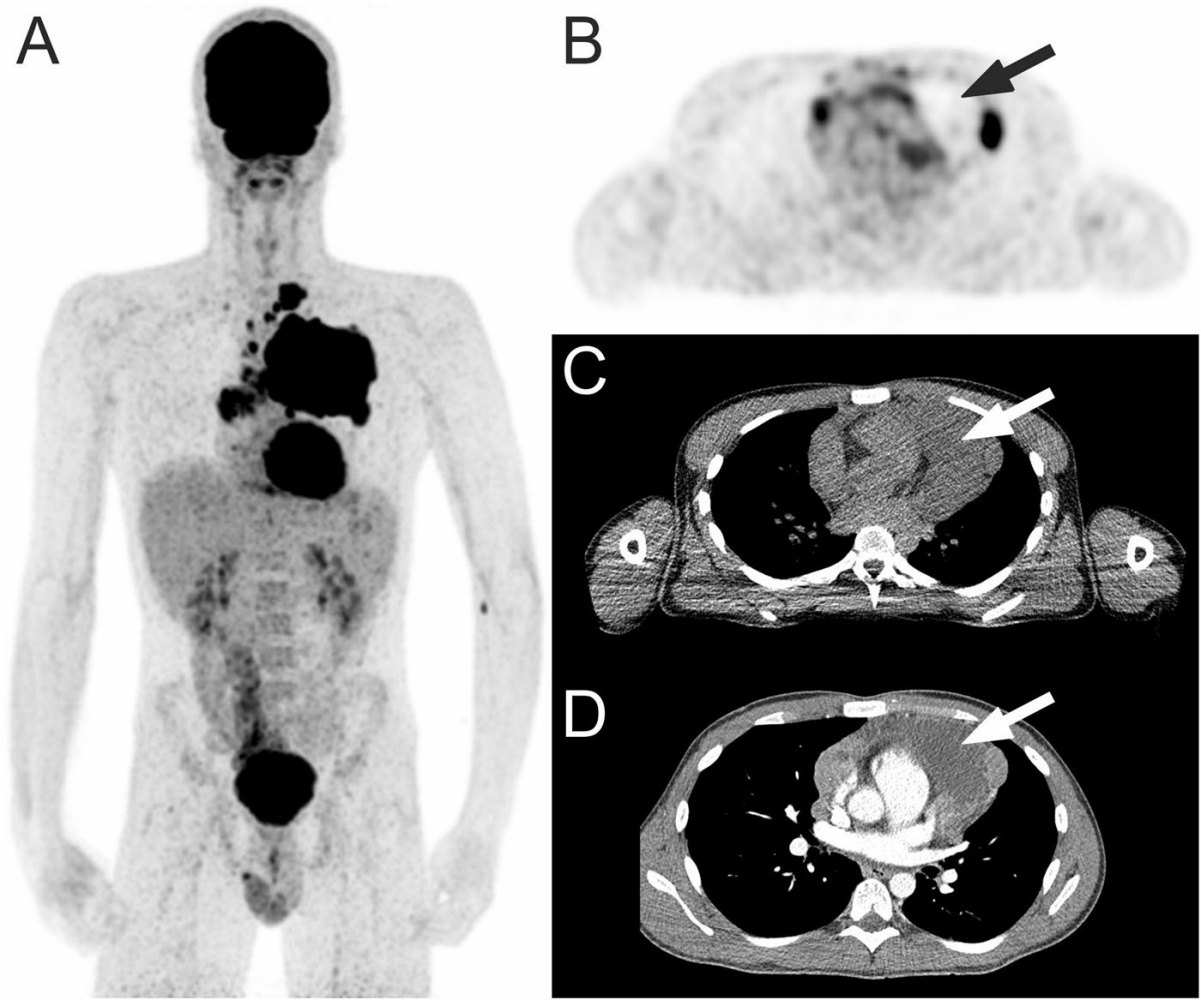
^18^F-FDG PET in a 21-year-old man with classical Hodgkin lymphoma. Coronal maximum intensity projection ^18^F-FDG PET shows a mediastinal tumor mass (**A**). Axial ^18^F-FDG PET with concomitant low-dose CT shows necrosis^visual^ with a photopenic area in the mediastinal tumor (**B**,**C**, arrows), with attenuation of around 18 HU on contrast-enhanced CT (**D**, arrow).

^18^F-FDG PET and CT review assessment of necrosis^visual^ was performed by an experienced reader (TCK) who was blinded to follow-up findings, including patient outcome, as previously described^41,42^. Briefly, all ^18^F-FDG PET and CT scans were visually assessed for the presence of tumor necrosis, within any nodal or extranodal ^18^F -FDG-avid lymphomatous lesion. Necrosis^visual^ was considered present if there were photopenic tumor areas on ^18^F-FDG PET and/or tumor areas with attenuation values between 10 and 30 Hounsfield units (HUs) on CT (Fig. 2).

### Statistical analysis

Baseline characteristics and semi quantitative parameters were described according to the presence of necrosis^visual^. Categorical variables were expressed as counts and percentages. Differences between two nominal variables were evaluated using Pearson’s Chi square or Fisher’s exact test (for expected counts ≤ 5). Continuous variables were expressed as median with range. Univariate logistic regression analyses were used to determine the association of established prognostic parameters (stage, IPS, bulky disease) and semiquantitative parameters (SUV_max_, SUV_mean_, SUV_peak_, MTV and TLG) with the presence of necrosis^visual^. For survival analysis progression free survival (PFS) and overall survival (OS) were used as endpoints. PFS was defined as the time from diagnosis until death, histologically confirmed relapse or progression of disease (defined as increased diameter of lesions on CT in combination with higher FDG-signal on PET or increased PET signal with increased serum TARC levels)^43,44^, whichever came first. OS was defined as time from diagnosis until death (from any cause). Surviving patients were censored at the last date of follow-up. Survival curves were estimated according to the Kaplan-Meier method. Cox proportional hazards model was used for univariate and bivariate survival analysis and results were reported as hazard ratio (HR), 95% confidence interval (CI) and *P*-value based on statistical Wald-test. A *P*-value of less than 0.05 indicated statistical significance. Continuous parameters such as MTV and TLG were dichotomized using the statistic median. Determination of an ideal cut-off via receiver operating characteristic (ROC) analysis was deliberately avoided due to the retrospective character of this study and the lack of a validation cohort^19^. All analyses were performed using R version 3.4.1 and R-studio version 1.0.153 software.

### Ethics approval and consent to participate

According to Dutch regulations, no medical ethical committee approval was required for this retrospective, observational study. This study utilized already registered/acquired medical information from patients, the use of which is regulated under the code for good clinical practice in the Netherlands and does not require informed consent in accordance with Dutch regulations. A waiver was obtained from the medical ethics committee of the UMCG on November 13th 2018.

## Results

### Baseline patient characteristics

The baseline characteristics of the 71 patients are summarized in Table 1. The median age across the entire patient population was 36.5 years with a range from 17–82 years. A majority of patients was treated with the ABVD regimen (n = 55, 77.5%).

**Table 1:** Baseline characteristics of patients with classical Hodgkin lymphoma according to necrosis^visual^ status as assessed by ^18^F-FDG PET and CT.

	Total (n = 71)		Necrosis^visual^ status				*P*-value
			Absent (n = 58)		Present (n = 13)		
	No.	%	No	%	No.	%	
**Gender**							0.31^a^
Male	43	*60*.*6*	33	*56*.*9*	10	*76*.*9*	
Female	28	*39*.*4*	25	*43*.*1*	3	*23*.*1*	
**Age**							0.74^b^
Median (range)	36 (17–82)		39 (17–82)		30 (19–68)		
Age ≤ 45 y	50	*70*.*4*	40	*69*.*0*	10	*76*.*9*	
Age > 45 y	21	*29*.*6*	18	*31*.*0*	3	*23*.*1*	
**Histology**							0.54^b^
NS	40	*56*.*3*	31	*53*.*4*	9	*69*.*2*	
MC	7	*9*.*9*	7	*12*.*1*	0	*0*.*0*	
LR	1	*1*.*4*	1	*1*.*7*	0	*0*.*0*	
NOS	23	*32*.*4*	19	*32*.*8*	4	*30*.*8*	
**EBV**^**†**^							0.71^b^
Negative	45	*63*.*4*	35	*60*.*3*	10	*76*.*9*	
Positive	14	*19*.*7*	12	*20*.*7*	2	*15*.*4*	
**B symptoms**							0.12^a^
No	33	*46*.*5*	30	*51*.*7*	3	*23*.*1*	
Yes	38	*53*.*5*	28	*48*.*3*	10	*76*.*9*	
**Treatment**							0.24^b^
ABVD	55	*77*.*5*	47	*81*.*0*	8	*61*.*5*	
BEACOPP	12	*16*.*9*	8	*13*.*8*	4	*30*.*8*	
CHOP	4	*5*.*6*	3	*5*.*2*	1	*7*.*7*	
**Radiotherapy**							1.0^a^
No	34	*47*.*9*	28	*48*.*3*	6	*46*.*2*	
Yes	37	*52*.*1*	30	*51*.*7*	7	*53*.*8*	

NS: nodular sclerosis; MC: mixed cellularity; LR: lymphocyte rich; NOS: not otherwise specified.

^a^Pearson’s Chi-squared test with Yates’ continuity correction.

^b^Fischer’s exact test for count data.

^†^Missing in 12 cases.

### Semiquantitative parameters

**Figure 3:**
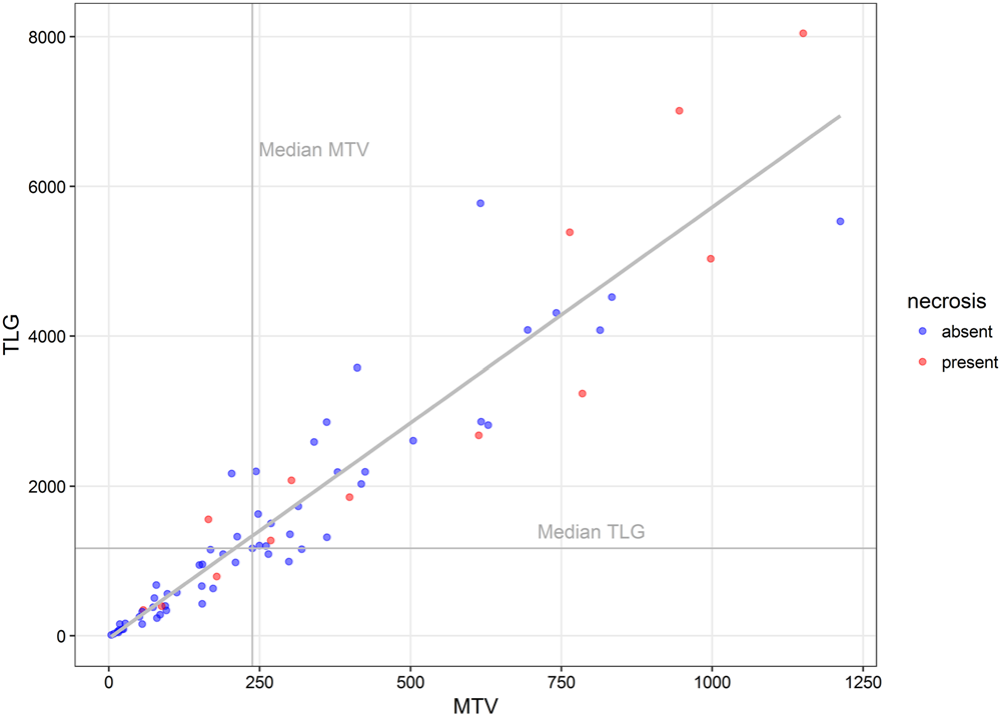
Dot plot showing the relation between metabolic tumor volume (MTV), total lesion glycolysis (TLG) and necrosis per investigated case (n = 71). Cases with necrosis^visual^ as determined by visual assessment of ^18^F-FDG PET scans and CT are depicted in red (n = 13).

The median SUV_max_, SUV_mean_ and SUV_peak_ were 7.1 (range 3.4–20.9), 4.2 (range 2.7–8.7) and 5.3 (range 2.7–17.1), respectively (Table 2). SUV parameters showed a skewed distribution, with the majority of values lying below the mean (mean SUV_max_ = 8.7; mean SUV_mean_ = 4.6; mean SUV_peak_ = 6.8). MTV (median = 237.8, range = 3.8–1212) and TLG (median = 1169, range = 12.1–8048) were highly variable among examined cases. Both MTV and TLG exhibited distributions skewed towards smaller values (mean MTV = 309.9 ml, mean TLG = 1747.7). The range of TLG became larger with greater volumes (MTV) (Fig. 3).

**Table 2:** Known prognostic parameters and semiquantitative measures according to necrosis^visual^ status.

	Total (n = 71)		Necrosis^visual^ status			
			Absent (n = 58)		Present (n = 13)	
	No.	%	No.	%	No.	%
**Stage**						
I-II	43	*60*.*6*	35	*60*.*3*	8	*61*.*5*
III-IV	28	*39*.*4*	23	*39*.*7*	5	*38*.*5*
**IPS**^†^						
0–2 (low risk)	50	*70*.*4*	42	*72*.*4*	8	*61*.*5*
3–7 (interm./high risk)	20	*28*.*2*	15	*25*.*9*	5	*38*.*5*
**Bulky disease**						
No	54	*76*.*1*	49	*84*.*5*	5	*38*.*5*
Yes	17	*23*.*9*	9	*15*.*5*	8	*61*.*5*
**SUV**_**max**_						
Median (range)	7.1 (3.4–20.9)		7.0 (3.4–21.0)		7.9 (5.4–18.3)	
**SUV**_**mean**_						
Median (range)	4.2 (2.7–8.7)		4.2 (2.7–8.7)		4.2 (3.3–8.2)	
**SUV**_**peak**_						
Median (range)	5.3 (2.7–17.1)		5.3 (2.7–17.1)		5.3 (4.1–15.7)	
**MTV**						
Median (range)	237.8 (3.8–1212)		206.7 (3.8–1212)		398.7 (56.9–1151)	
**TLG**						
Median (range)	1169 (12.1–8048)		1093 (12.1–5775)		2079 (346–8048)	

^†^Missing in 1 case.

### Necrosis

Necrosis^visual^ was observed in 13 of the 71 cases (18.3%). CT (n = 13; 100%) and ^18^F-FDG PET (n = 12; 92.3%) agreed on the presence of necrosis in 12 of the 13 cases (92.3%). In one case lesions with attenuation values of 10–30 HU were observed on CT, however photopenic areas in ^18^F-FDG PET could not be distinguished due to low resolution of the ^18^F-FDG PET images. Of the examined prognostic parameters, bulky disease (OR = 8.5; 95% CI: 2.3–32.1; *P* = 0.002) and TLG (OR = 4.3; 95% CI: 1.1–17.2; *P* = 0.041) correlated with a higher risk for necrosis^visual^ (Fig. 3), while stage and IPS did not. All other semiquantitative parameters were not associated with necrosis^visual^ (Table 3).

**Table 3:** Univariate logistic regression analyses for the association of investigated parameters with the presence of necrosis^visual^.

covariates	Odds ratio	95% CI	*P*-value (Wald-test)
**Stage**			
I-II	1		
III-IV	0.9	0.27–3.2	0.9
**IPS**			
0–2	1		
3–7	1.75	0.5–6.2	0.39
**Bulky disease**			
Absent	1		
Present	8.53	2.3–32.1	0.002**
**SUV**_**max**_			
<Median	1		
≥Median	1.13	0.34–3.8	0.85
**SUV**_**mean**_			
<Median	1		
≥Median	0.89	0.27–3.0	0.85
**SUV**_**peak**_			
<Median	1		
≥Median	0.77	0.23–2.6	0.67
**MTV**			
<Median	1		
≥Median	2.68	0.74–9.7	0.13
**TLG**			
<Median	1		
≥Median	4.3	1.1–17.2	0.041*

*P < 0.05.

**P < 0.01.

### Survival analysis

The median follow up was 42 months (95% CI; 34–60.7; IQR = 26.7–65.5). Twelve patients (16.9%) experienced an event as defined for PFS. Seven patients had a histologically confirmed relapse and 4 patients had progressive disease (two with evident increase in diameter (CT) and ^18^F-FDG signal (PET) and 2 with evident increase of ^18^F-FDG signal and subsequent increase in TARC levels). There were 2 treatment related deaths: One due to complications of first line therapy (death counted as PFS event), the other due to complications of salvage therapy (one of the 4 patients with progressive disease). Two patients died due to lymphoma. The 5-year PFS and OS for the entire cohort was 80.1% (95% CI: 69.9–91.8%) and 93.9% (95% CI: 88.3–99.9%), respectively. In univariate analysis for PFS IPS, bulky disease, MTV and TLG were prognostic factors, whereas necrosis was not (HR = 2.8; 95% CI: 0.8–9.4, *P* = 0.096) (Table 4). Apart from MTV (HR = 5.7; 95% CI: 1.3–26.2; *P* = 0.024) and TLG (HR = 5.5; 95% CI: 1.2–25; *P* = 0.028), all other semiquantitative parameters (SUV_max_, SUV_mean_, SUV_peak_) were not prognostic for PFS.

**Table 4:** Univariate Cox-regression analyses for progression free survival.

Prognostic factor	HR	95% CI	*P*-value (Wald-test)
**Stage**			
I-II	1		
III-IV	3.0	0.9–10.0	0.072
**IPS**			
0–2	1		
3–7	4.12	1.3–13.1	0.016*
**Bulky disease**			
Absent	1		
Present	5.3	1.7–16.8	0.005**
**Necrosis**^**visual**^			
Absent	1		
Present	2.8	0.8–9.4	0.096
**SUV**_**max**_			
<Median	1		
≥Median	0.7	0.2–2.1	0.49
**SUV**_**mean**_			
<Median	1		
≥Median	0.5	0.2–1.7	0.28
**SUV**_**peak**_			
<Median	1		
≥Median	0.7	0.2–2.1	0.49
**MTV**			
<Median	1		
≥Median	5.7	1.3–26.2	0.024*
**TLG**			
<Median	1		
≥Median	5.5	1.2–25.0	0.028*

*P < 0.05.

**P < 0.01.

## Discussion

In this retrospective study we performed a semiquantitative ^18^F-FDG PET analysis and assessment of necrosis (necrosis^visual^) by ^18^F-FDG PET and CT in cHL.

Necrosis^visual^ was observed in 18% of patients. Despite the additional use of ^18^F-FDG PET imaging the observed rate of necrosis^visual^ in the current study was slightly lower than the incidence reported by Hopper *et al*.^45^. This might be explained by the focus on thoracic lesions in the latter study. Since there was a 92% agreement between CT and ^18^F-FDG PET to detect necrosis^visual^ we cannot make any statement about the superiority of one modality over the other. Nonetheless, by using two independent modalities we can confirm that necrosis^visual^ is present in a significant part of the cHL population, despite the distinct pathophysiologic characteristics of cHL.

The majority of cells in cHL lesions are infiltrating immune cells which have different proliferative characteristics than cancer cells^46^. However, the immune cell infiltration in cHL is a pathological process that is abnormal and extensive in nature^46–48^. In the physiological setting lymph nodes harbor a wide spread capillary bed which provides nutrients and oxygen for the reticular meshwork in which immune cell interaction takes place^49^. In cHL, the infiltration by a substantial amount of immune cells frequently distorts and alters this highly vascularized lymph node architecture^50,51^. Nevertheless, biological evidence from studies investigating the role of the cHL microenvironment, suggests that mast cells, tumor associated macrophages, mesenchymal stromal cells as well as HRS cells themselves might be able to induce and contribute to angiogenesis^52^. In the light of this evidence, one might hypothesize that differences in proliferative activity between individuals, as well as an imbalance between proliferative activity of HRS cells and the infiltrate within individuals might explain why we observed necrosis^visual^ in almost 1 out of 5 patients in our cohort.

Parameters derived from semiquantitative analysis of ^18^F-FDG PET scans have been suggested as potential markers in lymphomas^53,54^. We could confirm these findings, as baseline MTV and TLG were prognostic for PFS in univariate survival analysis. Several small studies have implied that MTV can be used as a prognostic marker for survival in cHL^16,55–57^. In addition, previous investigations have suggested baseline TLG as a potentially useful prognostic marker for survival in cHL^20,54^ and other lymphomas^58–63^. However, as mentioned earlier, inconsistent results^19,64^, the challenge of standardizing quantification^65,66^, as well as small study cohorts, raise doubts about the potential of MTV measurements in clinical practice. While in NHL attempts are being made to include standardized semiquantitative analysis in prospective trials^67^, larger, prospective studies with standardized guidelines for semiquantitative analysis and appropriate validation cohorts for the determination of relevant cut-off values are needed to conclusively assess the role of semiquantitative measures in cHL^19,57^.

Our analyses demonstrate that necrosis^visual^ had no prognostic impact on PFSs and was strongly determined by the presence of bulky disease and TLG measures above the median. Since TLG had a significant impact on the presence of necrosis while MTV and SUV_mean_ did not, this points at a group of individual cases in which the interplay between volume and metabolic activity determines the development of necrosis.

The concept of tumor necrosis as a prognostic marker is based on the notion that it can develop when tumors outgrow their metabolic supply^68^. The correlation between proliferation rate and tumor necrosis, as exhibited by solid tumors such as clear cell renal cell carcinoma^69^ and nodular cutaneous melanomas^70^, is often stated to support this hypothesis. Necrosis as determined by visual assessment of ^18^F-FDG PET and CT scans, has been identified as an independent adverse prognostic factor in NHL^41,42,71,72^. However, conflicting reports^73,74^, as well as findings suggesting a crucial role for necrosis-induced inflammation^75,76^ imply necrosis as one of the causes for, rather than the result of tumor growth^77,78^. The relationship between necrosis and tumor volume might thus be very different in the setting of cHL with its distinct microenvironment, characterized by an extensive background of inflammatory infiltrate^79^.

This study has several limitations. Intrinsic to analyses of data from patients with cHL, there is heterogeneity with regard to received treatment (ABVD, escBEACOPP and, radiotherapy). It is important to point out that semiquantitative PET measures or the presence or absence of necrosis had no influence on treatment selection. Only 71 patients had ^18^F-FDG PET scans eligible for semiquantitative analysis, thereby reducing the power of the study. However, while our survival analysis is exploratory in nature we were able to reproduce the prognostic impact of IPS, bulky disease, MTV and TLG, reflecting a representative patient population. In the light of recent studies using ROC-analysis to find ideal cut offs for subsequent analysis, it is important to note that in the current study median values of semiquantitative ^18^F-FDG PET parameters were used for the dichotomization of continuous covariates. While we are aware that dichotomization of continuous variables always leads to a loss of information and reduction in power^80^, this option was chosen to avoid the introduction of arbitrary “optimal” cut-off values, which, in the absence of a validation cohort, can often be too specific for the retrospectively analyzed patient population, leading to unwarranted conclusions^19^.

## Conclusion

In this retrospective cohort study necrosis^visual^, as assessed by ^18^F-FDG PET and CT, was observed in 18% of cHL patients. Tumor necrosis^visual^ was significantly associated with bulky disease and TLG. In exploratory survival analysis the semiquantitative ^18^F-FDG PET parameters TLG and MTV were prognostic with regard to PFS, while necrosis^visual^ was not. Additional research is required to investigate the biological and clinical implications of tumor necrosis in cHL. While our results suggest no significant prognostic impact for necrosis^visual^, the role of semiquantitative ^18^F-FDG PET parameters should be validated in prospective studies.

## Supplementary information

Supplementary table 1 and 2

## Data Availability

All data generated or analyzed are in the current manuscript.
