# Supplementary material for: Relationship between semiquantitative 18F-fluorodeoxyglucose positron emission tomography metrics and necrosis in classical Hodgkin lymphoma
Source: Sci Rep. 2019 Jul 30;9:11073. doi: 10.1038/s41598-019-47453-5 (PMC6667466; doi:10.1038/s41598-019-47453-5)
Supplement: Supplementary file 1 — Supplementary table 1 and 2 [file 41598_2019_47453_MOESM1_ESM.pdf]

Relationship between semiquantitative ^{18}F-fluorodeoxyglucose positron emission tomography metrics and necrosis in classical Hodgkin lymphoma

X. U. Kahle*[1], F. M. Montes de Jesus[2], T. C. Kwee[3], T. van Meerten[1], A. Diepstra[4], S. Rosati[4], A. W. J. M. Glaudemans[2], W. Noordzij[2], W. J. Plattel[1], M. Nijland[1]

[1] Department of Hematology, University of Groningen, University Medical Center Groningen, The Netherlands

[2] Department of Nuclear Medicine and Molecular Imaging, University of Groningen, University Medical Center Groningen, The Netherlands

[3] Department of Radiology, University of Groningen, University Medical Center Groningen, The Netherlands

[4] Department of Pathology and Medical Biology, University of Groningen, University Medical Center Groningen, The Netherlands

***Corresponding / first author:**
Xaver U. Kahle
Department of Hematology, University Medical Center Groningen,
Hanzeplein 1, P.O. Box 30 001, 9700RB Groningen, The Netherlands
Phone: 0031-50-3616161
Fax: 0031-50-3618051
E-mail: x.kahle@umcg.nl

Running title: Semiquantitative PET metrics and necrosis in cHL

Keywords: classical Hodgkin lymphoma, ^{18}F-FDG PET, CT, necrosis, MTV, TLG

Tables: 1-4, supplementary tables 1 and 2

Figures: 1-3

Word count (main body): 2979

Abstract word count: 198

Supplementary table 1. ESMO guidelines 2014[1]: Risk factors

Large mediastinal mass (>1/3 of maximum horizontal chest diameter)
Age (≥ 50 years)
Elevated ESR (>50 mm/h without B symptoms, >30 mm/h with B symptoms)
≥4 nodal areas

Supplementary table 2. ESMO guidelines 2014[1] : Treatment

Limited	Limited stage with risk factors	Advanced stage
2/3 cycles of ABVD	*4 cycles of ABVD or 2 cycles of escBEACOPP + 2 cycles of ABVD (≤ 60 years)*	*6-8 cycles of ABVD or 6 cycles of escBEACOPP (≤ 60 years)*
20 Gy IFRT or ISRT	*30 Gy IFRT or ISRT*	*localized RT to residual lymphoma > 1.5cm (after ABVD) or localized RT to PET-positive residual lymphoma > 2.5 cm (after BEACOPP)*

References

1. Eichenauer, D. A. *et al.* Hodgkin's lymphoma: ESMO Clinical Practice Guidelines for diagnosis, treatment and follow-up. *Ann. Oncol.* **25,** iii70-iii75 (2014).
